# Factors Affecting Vocalization in Tengmalm’s Owl (*Aegolius funereus*) Fledglings during Post-Fledging Dependence Period: Scramble Competition or Honest Signalling of Need?

**DOI:** 10.1371/journal.pone.0095594

**Published:** 2014-04-23

**Authors:** Marek Kouba, Luděk Bartoš, Karel Št‚astný

**Affiliations:** 1 Czech University of Life Sciences Prague, Faculty of Environmental Sciences, Department of Ecology, Praha 6– Suchdol, Czech Republic; 2 Czech University of Life Sciences Prague, Faculty of Agrobiology, Food and Natural Resources, Department of Animal Science and Ethology, Praha 6– Suchdol, Czech Republic; 3 Institute of Animal Science, Department of Ethology, Praha 10– Uhříněves, Czech Republic; University of Lausanne, Switzerland

## Abstract

Begging behaviour of nestlings has been intensively studied for several decades as a key component of parent-offspring conflict. There are essentially two main theories to account for intensity of food solicitation among offspring: that intensity of begging is related to some form of scramble competition between nest mates or that it offers honest signalling of need to parents. The vast majority of studies which have addressed begging behaviour have been based on observations of, and experiments on, nestlings and have not considered begging behaviour, during the post-fledging period. Begging vocalizations in this post-fledging phase of dependence have rarely been studied, despite the importance of vocalizations as a communication method between offspring and parents, particularly for nocturnal species. We radiotracked 39 fledglings of the Tengmalm’s owl (*Aegolius funereus*) in two years with different availability of prey: 2010 (n = 29 fledglings) and 2011 (n = 10 fledglings) and made 1320 nightly localizations in which we recorded presence or absence of begging calls. Within years, the most important measures related to the probability of vocalization were body condition at fledging, time of night, number of surviving siblings, age and weather conditions. Begging intensity increased with age in both years; however, in the year with low prey availability fledglings vocalized significantly more often. The main factor causing these differences between years was probably the different availability of prey, affecting breeding success, post-fledging behaviour, and thus also both short- and long-term needs of offspring. We believe that our results suggest honest signalling of their fledgling’s need.

## Introduction

Begging behaviour as a major component within parent-offspring conflict (POC) has been studied in many avian species. Offspring commonly use both auditory (begging calls) and visual signals (coloured mouths, stretching necks and beaks, wing shaking) to obtain food from their parents. Begging behaviour has been used as a model to study POC and the evolution of signalling [1]. Theory predicts that family members are in conflict over the amount of parental investment provided, with offspring requesting more resources than parent are willing to provide [2], [3]. For parents, every investment in the current offspring which exceeds the optimum may be costly in terms of future reproduction and survival [4], [5]. According to POC theory, offspring should be selected to exaggerate their begging in order to manipulate parents and in order to get more resources than nest mates [2]. Thus POC can lead to the evolution of conspicuous begging [5], [6]. However, according to this theory begging behaviour should also be costly, or otherwise it would be evolutionary unstable; these costs, as reviewed by Roulin [7], might potentially be enhanced by risk of predation (loud begging attracts predators), punishment (begging may elicit aggressive behaviour from parents) and physical cost (begging may be energetically costly).

Since this recognition that there is a conflict between parents and offspring over the amount of parental investment, and that begging behaviour is one of the key elements in the POC, there has been wide-ranging debate as to whether begging displays are the outcome of scramble competition among siblings or are honest signals of offspring need [8]. Support is growing for both models, of signalling need towards parents (honest signalling: [5], [6]) and of competitive signalling between siblings (scramble competition: [9]–[12]). However, a review by Royle et al. [8] concluded that honesty may be context dependent and that begging may be honest only when the potential for conflict is low and food is not limiting because then neither party (parents and/or offspring) gains much by dishonesty.

The majority of theoretical and empirical studies of avian begging behaviour have focused on nestlings, due to the difficulties in observing juveniles once they have left the nest. Studies regarding begging by fledglings are, by comparison, rare, despite the fact that young birds continue to beg long after nest departure, usually till the end of the post-fledging dependence period (PFDP). Middleton et al. [13] found that, in American dippers (*Cinclus mexicanus*), fledglings begged at higher intensities in a year with lower food abundance and observed reduced parental provisioning rates during this year, suggesting that begging may reflect long-term condition and need (the total investment that a chick requires over the nestling phase or PFDP; see also [14]). Thompson et al. [15] showed that Pied babbler (*Turdoides bicolor*) fledglings revealed their need by moving to a riskier location, and were able to manipulate adults to achieve higher provisioning rates, with this providing support for the “blackmail theory” of Zahavi [16]. (This theory suggests that conspicuous solicitation may offer a mechanism for young to “blackmail” carers into provisioning them, by threatening their own destruction).

The Tengmalm’s owl (*Aegolius funereus*) is a small, nocturnal, cavity-nesting owl (male body mass ca. 100 g), living in coniferous forests in the boreal zone and in alpine forests further south in Eurasia [17]; it feeds mainly on small mammals [18]–[21]. Hatching occurs at approximately two-day intervals [22]. The young stay in the nest for 27–38 days after hatching [18], [23]–[25], thus fledging at different times, and reach independence 5–9 weeks after fledging [18], [26]–[29]. The great majority of prey brought to the young throughout the late nestling and PFDP, in this particular species, is delivered by the male [27], [28], [30]. During this time the offspring vocalize to solicit food from parents with short, hissing *cheet* calls [31].

In this paper we explore vocalizations of fledglings throughout the PFDP, in order first to confirm the presumption that fledglings’ vocalization is a manifestation of auditory begging behaviour to solicit food from parents as suggested by König & Weick [31], and then to resolve whether such calls relate more to an expression of competition between siblings, or reflect an honest signal to the parent of need.

We predicted that

i) if vocalizations do indeed act as begging signals, individuals which could be seen to have received prey should not vocalize, in contrast to those without food.

ii) if the fledglings’ vocalization is an honest signal to the feeding parent that the fledgling is hungry, rather than indicating scramble competition between siblings, then fledglings in better condition (indicated by either longer wing length or higher body weight; e.g., [32], [33]) should be found to vocalize less often than individuals in poor body condition, and iii) begging should decrease through the night, since the parents do not hunt during daylight [23], [31] and thus the chicks will be hungrier early in the night, having fasted through the day.

If indeed begging vocalizations relate to need rather than simple competition, we also predicted that:

iv) the probability of begging should increase with increasing brood size because it will take longer to satiate all individuals.

By the same logic we predicted that v) fledglings will vocalize more often in years with low prey availability because it will be more difficult for the parents to keep them satiated (see for example [13]).

Finally, we also predicted that the frequency of vocalizations

vi) will increase with age of the fledglings as reported for Eagle owl (*Bubo bubo*) fledglings [34] and

vii) will be lower during harsh weather, especially during rainy nights and/or strong wind because Klaus et al. [35] noticed that as little as 5 mm of rain caused a decrease in nest feeding visits in Tengmalm’s owl.

## Materials and Methods

### Study Area

The study was carried out during two breeding seasons 2010–2011 in an area close to the water reservoir Fláje in the Ore Mountains, Czech Republic (50° 40′ N, 13° 35′ E). This area (75 km^2^, 730–960 m a. s. l.) is now largely forested, with the predominant species being Blue spruce (*Picea pungens*, occupying approximately 28% of the study area), Norway spruce (*Picea abies*, 26%), Birch (*Betula* sp., 11%), European mountain ash (*Sorbus aucuparia*, 5%), European beech (*Fagus sylvatica*, 4%) and European larch (*Larix decidua*, 4%). Outside the forested parts the vegetation is dominated by Wood reeds (*Calamagrostis villosa*) and solitary European beech [36]. To compensate for the lack of natural tree cavities, 170 wooden nestboxes lined with wood chips (with the base 25×25 cm, height 40 cm and with an entrance hole 8 cm in diameter) have been installed gradually in the area since 1999.

Weather data were obtained from the closest weather stations to the study area. The average daily temperature (°C) and wind speed (m/s) were taken from the station in Nová Ves v Horách, located ca. 5 km from the study area. Daily precipitation (mm) was taken from the station in Český Jiřetín, located ca. 1.5 km from the study area.

### Field Procedures

Following the method of Eldegard & Sonerud [26], all nestboxes were visited weekly by one of the authors (MK) from early March to find nests, and thereafter sufficiently often to check number of eggs and hatchlings and to determine exact hatching date (±1 day). From 25 days after hatching of the first chick, (i.e., shortly before that time when chicks were expected to leave the nestbox), the nestboxes were checked at one or two-day-intervals. All individuals were weighed and the length of wing was measured to estimate the appropriate time for tagging and to get data on individual body condition [18], [32], [33], [37].

Following the method of Hipkiss & Hörnfeldt [38] a 50 µl blood sample was taken from each nestling by brachial vein puncture under the wing, ca. 14 days after hatching, for molecular sexing. Sex determination of nestlings relied on polymerase chain reaction (PCR) amplification of one intron from the sex chromosome linked *CHD1* gene, which in birds differs in size between the Z and W chromosomes [39]. Males showed only the shorter Z-fragment, while females were characterised by displaying both a 1.2 kb W-specific and a 0.7 kb Z-specific fragment [39]. Owls were trapped, handled and tagged under permit No. 530/758 R/08-Abt/UL from the Ministry of the Environment of the Czech Republic, were ringed under the Ringing Centre of the National Museum in Prague permit No. 329; all efforts were made to minimize suffering.

Fledglings from six nestboxes in 2010 (n = 29) and from five nestboxes in 2011 (n = 10) were equipped with leg-mount transmitter type PIP4 (Biotrack Ltd., UK) about four days before fledging [29]. Transmitters weighed 2.3 g in 2010 and 2.0 g in 2011 (lifespan ±10 weeks) which followed welfare recommendations not to exceed 3% of body weight of tagged individuals (e.g., [40]). Specifically, transmitters were respectively 1.84% and 1.76% of fledgling’s body weight on average, in 2010 and 2011.

Thereafter, nestboxes were visited at 12–hour-intervals during the night (22∶00–04∶00) and during daylight (10∶00–21∶00) until all siblings had fledged and we could determine the exact date of nestbox departure. After fledging, the young were located once every night by the ‘homing-in’ method [41] till they became independent (i.e., we followed the signal to a particular tree or until we saw the individual; it seemed our presence did not disturb either begging or silent individuals). Radio signals were received by using a MVT-9000 receiver (Yupiteru Industries Co. Ltd., Japan) and 3-element Yagi antenna. Fledglings’ positions (n = 1320 locations in total) were recorded using the GPS receiver (Garmin GPSmap 60CSx) and, during and after homing in on the young, we recorded presence or absence of vocalization for all fledglings. Thus, we recorded vocalization (presence or absence) during 10 minutes interval every night for each individual from fledging till the end of PFDP as in the study by Pedersen et al. [42]; (data were collected during the same time periods in both years). We defined the end of PFDP with the first rapid and abrupt movement away from habitual locations (after [43] and see [29]), which may correspond with cessation of begging for food [44].

Prey availability in the study area assessed by snap-trapping was 18.5 times higher in 2010 than in 2011 [25], [29].

### Statistical Analyses

All data were analysed with the aid of SAS System version 9.3 (SAS Institute Inc.). The analysis was made in three steps. Firstly, using the t-test, we compared fledglings’ body weight and wing length between seasons. Secondly, to check for possible multicollinearity we calculated correlations between the individual variables involved (listed in Table 1). Correlation was found between wing length and body weight (0.61, P<0.0001), wing length and number of present siblings (0.41, P<0.0001), and wing length and time of night when each individual fledgling was located (0.44, P<0.0001). We subsequently made a judgment of the extent of collinearity by checking related statistics, such as tolerance value or variance inflation factor (VIF), Eigenvalue, and condition number following the approach of Belsley et al. [45] and using TOL, VIF and COLLIN options of the MODEL statement in the SAS REG procedure. Low eigenvalues and large condition indices indicated that the variables – body weight, mean daily temperature and mean wind speed – were possibly redundant and therefore we omitted these variables from the further analyses.

**Table 1:** Fixed effects used in the GLMM 1, GLMM 2, and GLMM 3 for the probability of vocalization by radiotracked fledglings (29 in 2010 and 10 in 2011).

Fixed effect	2010	2011
Date of hatching	23 April–10 May	12 April–7 May
Date of fledging	23 May–15 June	14 May–10 June
Date of reaching independence	5–30 July	11 July–3 August
Body weight at fledging	range: 95–152 g	range: 85–134 g
	Mean ± SD 125±13 g	Mean ± SD 114±16 g^1^
Wing length approximated to the age of 30 days from hatching	range: 99–153 mm	range: 103–134 mm
	Mean ± SD 129±13 mm	Mean ± SD 115±10 mm^2^
Sex of individuals	10∶19–F : M	4∶6– F : M
Number of fledged siblings^3^	3–8	1–4
Number of present siblings^4^	1–7	1–3
Maximal number of siblings seen outside the nestbox^5^	3–7	1–3
Duration of period within the nestbox from hatching	28–36 days	27–38 days
Individual duration of PFDP	34–51 days	53–61 days
Time from hatching (fledgling age)	28–87 days	27–98 days
Individual fledgling home range size throughout the PFDP^6^	5.3–61.1 ha	11.9–97.1 ha
Pooled sibling home range size throughout the PFDP^7^	10.2–73.2 ha	11.9–101.9 ha
Maximal fledgling distance from the nestbox throughout the PFDP	273–1334 m	958–2004 m
Mortality rate within the sibling flock^8^	0–25%	0–100%
Time of night when each individual fledgling was located	22∶00–3∶59 hh∶mm	22∶00–3∶43 hh∶mm
Mean daily temperature^9^	6.6–25°C	5.9–20°C
Mean wind speed^10^	0.7–8.0 m/s	1.0–8.7 m/s
Daily precipitation^11^	0–62 mm	0–36 mm

1 Difference in body weight between years t = 2.31, p = 0.0265.

2 Difference in measured wing length between years t = 3.13, p = 0.0034.

3 Total number of individuals fledged from one individual nestbox.

4 Actual number of individuals still alive seen outside the nestbox on any given day.

5 Maximal number of live individuals seen outside the nestbox after all young had fledged.

6, 7 Individual fledgling and pooled sibling home range sizes (data from all siblings from particular nestbox) throughout the PFDP were established by minimum convex polygon method [64].

8 Number of dead fledglings as percentage of the total number of fledglings.

9, 10, 11 Log-transformed for the analyses.

Resolution of the dependency observed between wing length and number of siblings present by use of principal components analysis, as generally recommended, was not an option for us because we needed to estimate the effect of both variables. Therefore, we decided to apply two different models (GLMM 1 and GLMM 2) in the next step, with each model containing one or the other variable, but not both. However, since all the fixed effects were in fact also statistically significant (and the inter-correlation coefficients did not seem to be prohibitively large for them to be included alongside in the same model), we apply also GLMM 3 containing all the variables mentioned above. Another support for including also the third model was the fact that the Estimate values and SE of Estimate values were similar in all of the different models (Table S1), thus, giving us the ultimate argument for multicollinearity not being a problem in the final models. The meteorological variables (precipitation, temperature, and wind speed) were highly intercorrelated. Hence, we used the only variable, the precipitation, for further analyses.

In the third step we tested the probability of vocalization by the Tengmalm’s owl fledglings and the association between this and other factors, using a General Linear Mixed Models (GLMM, PROC GLIMMIX for binary distribution). Link function was logit and error terms were binomial in the GLMMs. To account for the use of repeated measures on the same individuals from the same nestbox, analyses were performed using mixed model analyses with individual fledgling nested within nesting box as a random effect. Fixed effects employed within all three models are summarised in Table 1. Since we expected significant differences between the years (see [29] and Table 1), all fixed effects were entered into all three models nested within the year (2010 and 2011).

We constructed the GLMMs by entering first those factors expected to have an effect on frequency of vocalization (GLMM 1: wing length, time from hatching and log-transformed daily precipitation; GLMM 2: number of siblings present, time from hatching and time of night when each individual fledgling was located; GLMM 3 contained all factors stated in the two previous models) and then checking all three models with addition of the factors which could also affect the result. The significance of each fixed effect in the GLMMs was assessed by the F-test. Any factors which did not add to significance (P>0.05) were dropped from the model and will not be mentioned any further. Where appropriate we tested interaction terms.

Associations between the dependent variable and fixed effects are presented as logit (the log of an odds [46]) plotted against the fixed effect with predicted regression lines for each year.

## Results

Body weight and wing length were both consistently larger in 2010 in comparison to 2011 (Table 1). During 2010 fledglings were often silent and continuous use of the radio-receiver to locate them was necessary on nearly every occasion. In contrast, fledglings called constantly almost every night during 2011. Radioequipment was thus in many cases used just for determination of general direction of the fledglings, thereafter locating them by sound in order to get close to them, finally identifying individual fledglings again by radioequipment. In neither season were fledgling vocalizations common during the first five days after fledging (only 7 out of 128 fledglings located in 2010 and 6 of 39 in 2011).

**Figure 1:**
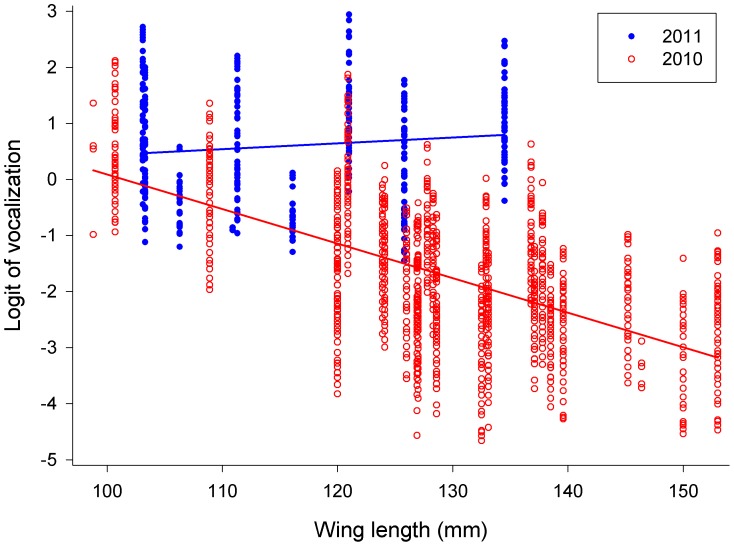
Logit of vocalization plotted against the length of wing approximated to the age of 30 days from hatching in 2010 (open red circles) and 2011 (filled blue circles).

**Figure 2:**
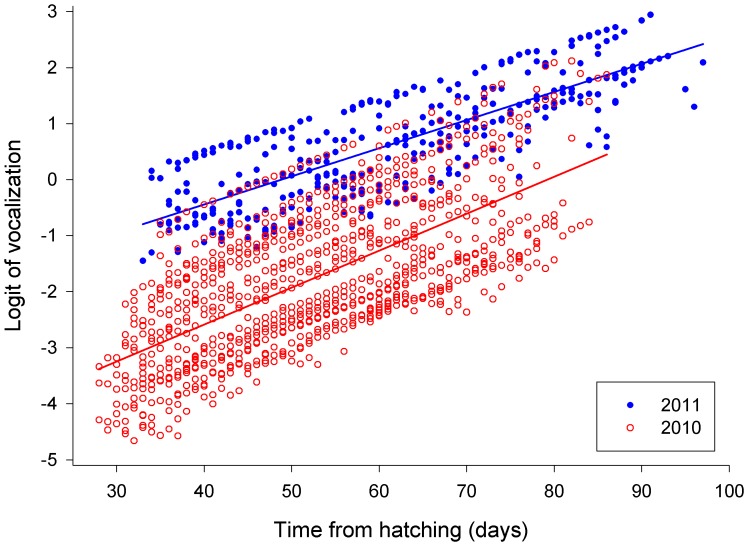
Logit of vocalization plotted against the owlets age from hatching for 2010 (open red circles) and 2011 (filled blue circles).

**Figure 3:**
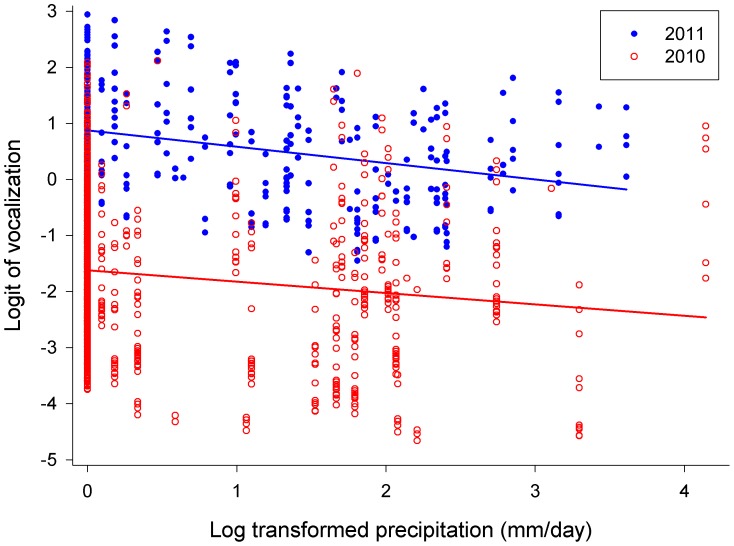
Logit of vocalization plotted against the log-transformed daily precipitation in 2010 (open red circles) and 2011 (filled blue circles).

Fledglings which, when located, were found to have a prey item (n = 31 cases; 21 in 2010 and 10 in 2011) were silent in 30 cases; in one case only (in 2011), a singleton who had just received a prey item continued to vocalize a few more minutes thereafter. Results of the GLMM 1 (including wing length as a fixed factor but not number of siblings; Table 2) revealed that the probability of vocalization was dependent on wing length at fledging, nested within the year (Fig. 1; ii), age from hatching, nested within the year (Fig. 2; vi), and log-transformed daily precipitation, nested within the year (Fig. 3; vii).

**Table 2:** The results of the GLMM 1, GLMM 2 and, GLMM 3 for factors affecting the probability of vocalization by Tengmalm’s owl fledglings throughout the PFDP.

Fixed effect – GLMM 1	Num DF	Den DF	F value	P =
Wing length approximated to the age of 30 days from hatching nested within the year	2	60.44	10.69	0.0001
Time from hatching nested within the year	2	1309	49.92	0.0001
Log-transformed daily precipitation nested within the year	2	1309	5.62	0.0037
**Fixed effect – GLMM 2**	**Num DF**	**Den DF**	**F value**	**P = **
Time of night when each individual fledgling was located nested within the year	2	182	8.69	0.0002
Time from hatching nested within the year	2	1309	52.8	0.0001
Number of present siblings nested within the year	2	57.69	3.1	0.0526
**Fixed effect – GLMM 3**	**Num DF**	**Den DF**	**F value**	**P = **
Wing length approximated to the age of 30 days from hatching nested within the year	2	35.67	11.62	0.0001
Time of night when each individual fledgling was located nested within the year	2	167.3	12.09	0.0001
Log-transformed daily precipitation nested within the year	2	1305	6.38	0.0018
Time from hatching nested within the year	2	1305	44.55	0.0001
Number of present siblings nested within the year	2	50.66	5.02	0.0103

Probability of vocalization under this model was negatively related to the wing length at fledging in 2010 but positively dependent on wing length at fledging in 2011 (Fig. 1). Probability of vocalization increased with increasing age in both seasons (Fig. 2); daily precipitation affected vocalization negatively in both seasons (Fig. 3), and individuals located during the rain did not vocalize at all (not quantified).

**Figure 4:**
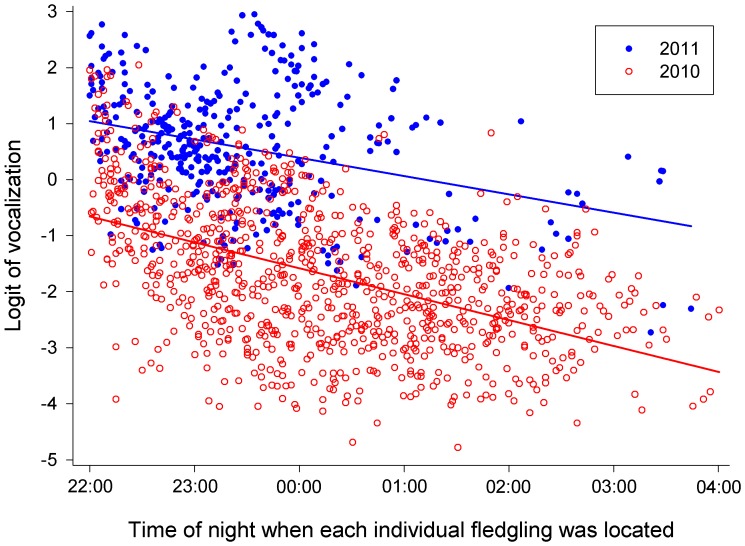
Logit of vocalization plotted against the time of night when fledglings were located, in 2010 (open red circles) and 2011 (filled blue circles).

**Figure 5:**
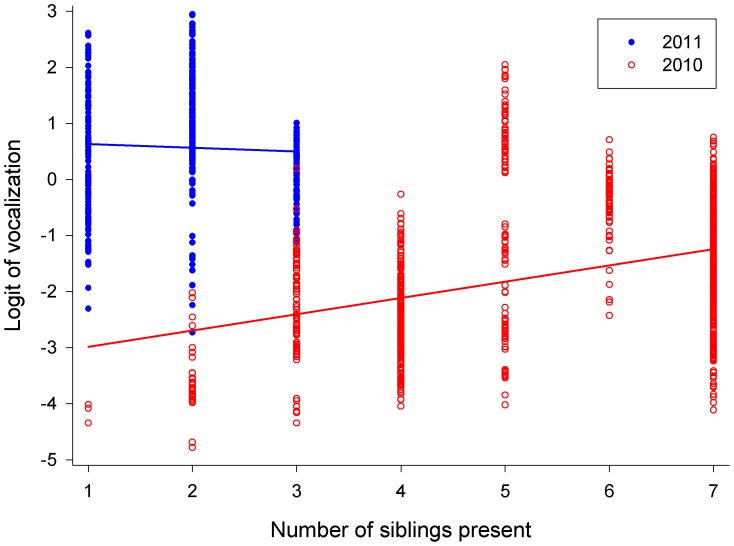
Logit of vocalization plotted against the number of siblings present for 2010 (open red circles) and 2011 (filled blue circles).

Results of the GLMM 2 (including number of siblings present, but excluding wing length; Table 2) revealed that the probability of vocalization was dependent on time of night when each individual fledgling was located, nested within the year (Fig. 4; iii), age from hatching, nested within the year (very similar to Fig. 2 and therefore not shown; vi), and number of siblings present, nested within the year (Fig. 5; iv). Although the effect of number of siblings present did not reach formal level of significance, we left it in the model which was then offered a better fit as regards to Akaike’s, Schwarz’s and a finite-sample corrected Akaike Information Criterion.

Probability of vocalization under this model was negatively associated with time of night in both seasons (Fig. 4). Vocalization increased with increasing age in both seasons (very similar to Fig. 2 and therefore not shown), and was positively related to the number of siblings present in 2010 but negatively dependent on sibling number in 2011 (Fig. 5).

Results of the GLMM 3 were virtually identical with the two previous models regarding every single fixed effect (Table 2); also all figures for this third model were very similar to figures presented in the first and second model (Fig. 1–5) and therefore are not shown.

In the year with poor food availability (2011), three of five broods monitored consisted only of a single chick. These singletons begged for food intensively (with a frequency similar to that of siblings from other two nestboxes) although they had no sib-competitors; by contrast individuals in the numerous sib-flocks (3–7 siblings) monitored in the more food-rich year (2010) begged with far lower intensity.

## Discussion

So far as we know, this is the first study evaluating vocalization behaviour in fledgling owls during two successive years with different prey availability. In terms of our initial predictions, we found support for vocalization as representing begging for food. The higher incidence of vocalizations recorded in 2011 (even with smaller number of siblings) suggested that fledglings received less prey than in 2010 [29]; this together with the fact that fledglings located with prey items did not vocalize does suggest that the main function of vocalizations is indeed begging for food (prediction i).

We could not directly test the relationship between the probability of vocalization and prey availability, but because we have demonstrated difference between years, we may speculate that this difference was indeed a result of different prey availability.

Middleton et al. [13] found that in American dippers, fledglings begged at higher intensities in a year with lower food abundance and they observed reduced parental provisioning rates during this year, suggesting that begging may reflect long-term condition. This fits with our results showing the effect of wing length at fledging on probability to vocalize (Fig. 1). In the year of poor food availability, begging intensity increased with the length of wing and thus with better condition. This is especially interesting because it is in contrast to our original prediction (ii) and also to findings by Price et al. [14] and Quillfeldt [1] who showed (albeit pre-fledging) that nestlings in poor condition increased begging intensity. Perhaps our finding can be explained by the fact that fledglings which started off in better condition during the poor food year can afford to invest more energy in begging than chicks in poorer condition, and did so because during that food year they were never fully satiated. By contrast in the year with better food availability fledglings in better condition vocalize with significantly lower probability because they have a lesser requirement for food. Both hunger and condition have also been shown to influence begging intensity in other studies [32], [47], [48].

Body weight at fledging did not remain within the final model selected. Body weight in Tengmalm’s owl nestlings stabilizes at the age of approximately 25 days after hatching [18], [49] and, thereafter, it seems that fledglings’ body weight simply bounces back and forth, depending on when the young last fed. The fact that the interaction between body weight and wing length was not significant in the model is further suggestive that body weight is fluctuating over time. Thus, despite the fact that we recorded significant differences in individual body weight at fledging between years, this variable is not suitable for assessing long-term body condition. On the other hand, wing length continues to grow throughout the nestling period [18], [49], showing incomplete feather growth at fledging, and is thus much more suitable for assessing body condition. Moreover, wing length is the most important variable influencing duration of nestlings’ stay in the nestbox (Kouba, unpublished data). However, our findings that the young in the food-rich year were significantly heavier at fledging than in the poor food year are still in accordance with findings of Leonard & Horn [50], [51] that begging intensity increased with food deprivation and decreasing nestling weight. Increase in begging intensity as a result of food deprivation has also been shown by other studies [32], [52]–[54], on magpies (*Pica pica*), pigeons (*Columba livia*), Cory’s shearwater (*Calonectris diomedea*) and barn swallow (*Hirundo rustica*) nestlings, respectively. It seems that this begging call behaviour pattern reflects long-term need by offspring and supports thus the theory of honest signalling of need [5].

Probability of vocalizations decreased as the night progressed in both seasons (Fig. 4 and prediction iii) indicating that the fledglings were indeed hungry after long daylight hours regardless of the prey availability and the begging intensity decreases throughout the night as parents begin to feed and gradually satiate their chicks. This in itself may be considered further evidence in favour of begging calls as honest signalling [5] rather than scramble competition [9].

In neither season were fledgling vocalizations common during the first five days after fledging. We believe that fledglings were silent during this time most probably due to potential danger of predation by mammals, and thus, as an anti-predator response. Predation by mammals is common immediately after leaving the nest ([55], Kouba, unpublished data) and the young are most vulnerable during this time due to considerably incomplete feather growth and still poor flying.

In our study, probability of vocalization was also dependent on the number of siblings present (Fig. 5; prediction iv). In the season with high prey availability (2010) the probability of begging increased with the number of siblings present, which is consistent with our predictions and in accordance with studies on European starlings (*Sturnus vulgaris*) by Wright & Cuthill [56] and Tree swallow (*Tachycineta bicolor*) nestlings by Leonard et al. [57] which showed that begging intensity increased with brood size. While begging intensity may also increase without any influence of food deprivation or condition, when nestlings are stimulated to beg by the begging of nest mates [57], [58], we think this not applicable in relation to fledgling Tengmalm’s owls because they are moving up to tens of meters apart (maximum about 300 m), but still begging for food. We think that if begging calls were directed towards competition for food items, the owlets would position themselves close to each other and not call from rather more separated locations (Kouba, unpublished data).

In the year with poor food availability (2011) three of five broods monitored consisted only of a single chick. These singletons begged for food with a higher intensity than chicks during the year of higher food availability (prediction v), but there was no significant difference between intensity of calling by these singletons and calling by the siblings fledged from the other two nestboxes. Conversely, it seems that in the year of higher food abundance the amount of prey was not limiting (it was common to find several uneaten pieces of prey items in nestboxes after all individuals fledged); both observations suggest that begging calls signal honest need [5], [6], [8]. We suggest therefore that sibling competition may play only a minor role in calling by Tengmalm’s owl chicks during the PFDP.

Probability of vocalization also increased with age in both seasons (Fig. 2 and prediction vi) which is in good agreement with the trend found by Penteriani et al. [34] in young Eagle owls. In contrast, however, Pedersen et al. [42] reported that frequency and intensity of begging calls decreased with age in fledgling Little owls (*Athene noctua*). These different results may be due to differences in prey consumed by these species. While Little owls largely feed on a wide range of invertebrates during the summer [59] and their fledglings probably begin to hunt on their own, to fulfil part of their needs, within a few weeks after fledging [42], Tengmalm’s owls and Eagle owls are almost entirely dependent on vertebrate prey throughout the year (small mammals and birds; [18], and mammals and birds in size up to hares and herons; [31], respectively), and their fledglings do not start to hunt alone until around the end of PFDP. Thus, as the time of independence is approaching, fledglings of the Tengmalm’s and Eagle owl may escalate their begging call behaviour in order to maximize their own fitness (according to the theory of POC over parental care; [2]) especially because at this time parents may be tending to provide less food to their offspring [60], [61].

Finally, our results suggested that the frequency of calling decreased with increasing amount of precipitation in both seasons (Fig. 3 and prediction vii). We believe that this finding is connected with the hunting strategy of Tengmalm’s owl adults. They depend heavily on sound to localize ground-dwelling prey [62], [63] and are not able to hunt efficiently in rain; in consequence they do not bring prey items to their offspring during such periods. It is possible that fledglings somehow realize that their parents will not provide them with food during rains and/or storms, and thus, do not beg during these periods. In accordance with this Klaus et al. [35] described that in Germany nest feeding visits in Tengmalm’s owl decreased during rain, although Korpimäki [18] stated that weather did not affect visits of owls to nests in Finland.

To conclude: we believe that our findings regarding begging call behaviour patterns support the theory of begging calling as honest signal of need to parents (e.g., [5], [6]) rather than scramble competition (e.g., [9]). This is supported in particular by the fact that singletons begged for food frequently although they had no sib-competitors, by a decrease in begging intensity observed in both years with time of night, and by the fact that significantly higher begging intensity was recorded overall in the year with low prey availability compared to the year with high prey availability.

## Supporting Information

Table S1 Solution for fixed effects affecting the probability of vocalization by Tengmalm’s owl fledglings throughout the PFDP included in the GLMM 1, GLMM 2, and GLMM 3.
(DOC)
