# Supplementary material for: Factors Affecting Vocalization in Tengmalm’s Owl (Aegolius funereus) Fledglings during Post-Fledging Dependence Period: Scramble Competition or Honest Signalling of Need?
Source: PLoS One. 2014 Apr 23;9(4):e95594. doi: 10.1371/journal.pone.0095594 (PMC3997414; doi:10.1371/journal.pone.0095594)
Supplement: Table S1 — Solution for fixed effects affecting the probability of vocalization by Tengmalm’s owl fledglings throughout the PFDP included in the GLMM 1, GLMM 2, and GLMM 3. (DOC) [file pone.0095594.s001.doc]

**Table S1:** Solution for fixed effects affecting the probability of vocalization by Tengmalm’s owl fledglings throughout the PFDP included in the GLMM 1, GLMM 2, and GLMM 3.

| **Solutions for Fixed Effects – GLMM 1** | **year** | **Estimate** | **SE** | **DF** | **t value** | **P =** |
| --- | --- | --- | --- | --- | --- | --- |
| Intercept |  | 0.4087 | 1.8118 | 31.18 | 0.23 | 0.823 |
| Wing length approximated to the age of 30 days from hatching nested within the year | 2010 | -0.0412 | 0.01384 | 30.58 | -2.98 | 0.0056 |
| Wing length approximated to the age of 30 days from hatching nested within the year | 2011 | -0.02175 | 0.01608 | 31.91 | -1.35 | 0.1857 |
| Time from hatching nested within the year | 2010 | 0.06342 | 0.007748 | 1309 | 8.18 | 0.0001 |
| Time from hatching nested within the year | 2011 | 0.04767 | 0.008214 | 1309 | 5.8 | 0.0001 |
| Log-transformed daily precipitation nested within the year | 2010 | -0.2127 | 0.1053 | 1309 | -2.02 | 0.0435 |
| Log-transformed daily precipitation nested within the year | 2011 | -0.3054 | 0.1143 | 1309 | -2.67 | 0.0076 |
| **Solutions for Fixed Effects – GLMM 2** | **year** | **Estimate** | **SE** | **DF** | **t value** | **P =** |
| Intercept |  | 2.5814 | 1.9474 | 343.8 | 1.33 | 0.1859 |
| Time of night when each individual fledgling was located nested within the year | 2010 | -0.0001 | 0.000023 | 335.7 | -4.16 | 0.0001 |
| Time of night when each individual fledgling was located nested within the year | 2011 | -0.00008 | 0.000023 | 455.2 | -3.32 | 0.001 |
| Time from hatching nested within the year | 2010 | 0.06321 | 0.007858 | 1309 | 8.04 | 0.0001 |
| Time from hatching nested within the year | 2011 | 0.05326 | 0.008552 | 1309 | 6.23 | 0.0001 |
| Number of present siblings nested within the year | 2010 | 0.1425 | 0.1035 | 56.54 | 1.38 | 0.1738 |
| Number of present siblings nested within the year | 2011 | 0.6524 | 0.3098 | 58.78 | 2.11 | 0.0395 |
| **Solutions for Fixed Effects – GLMM 3** | **year** | **Estimate** | **SE** | **DF** | **t value** | **P =** |
| Intercept |  | 3.5413 | 2.2998 | 48.7 | 1.54 | 0.1301 |
| Wing length approximated to the age of 30 days from hatching nested within the year | 2010 | -0.0622 | 0.01584 | 35.95 | -3.93 | 0.0004 |
| Wing length approximated to the age of 30 days from hatching nested within the year | 2011 | 0.05172 | 0.02254 | 32.92 | 2.29 | 0.0283 |
| Time of night when each individual fledgling was located nested within the year | 2010 | -0.00002 | 0.000029 | 195.8 | -0.67 | 0.5034 |
| Time of night when each individual fledgling was located nested within the year | 2011 | -0.00016 | 0.000032 | 281.3 | -4.9 | 0.0001 |
| Log-transformed daily precipitation nested within the year | 2010 | -0.1807 | 0.1063 | 1305 | -1.7 | 0.0893 |
| Log-transformed daily precipitation nested within the year | 2011 | -0.3727 | 0.1186 | 1305 | -3.14 | 0.0017 |
| Time from hatching nested within the year | 2010 | 0.06021 | 0.008221 | 1305 | 7.32 | 0.0001 |
| Time from hatching nested within the year | 2011 | 0.05285 | 0.008931 | 1305 | 5.92 | 0.0001 |
| Number of present siblings nested within the year | 2010 | 0.2543 | 0.1088 | 45.68 | 2.34 | 0.0239 |
| Number of present siblings nested within the year | 2011 | 0.674 | 0.3072 | 56.71 | 2.19 | 0.0323 |
